# Supplementary material for: The genome sequence of the Corsican Dappled White, Euchloe insularis (Staudinger, 1861) (Lepidoptera: Pieridae)
Source: Wellcome Open Res. 2026 Sep 14;11:665. [Version 1] doi: 10.12688/wellcomeopenres.27820.1 (PMC13658996; doi:10.12688/wellcomeopenres.27820.1)
Supplement: Peer review history

# Reviewer report: Reviewer response for version 1

**DOI:** 10.21956/wellcomeopenres.30613.r171689

**Published:** 2026-10-12

**Authors:** Jurate De Prins

**Affiliations:** 1 Flemish Entomological Society, Vrieselhof, Belgium

As the authors very rightly state, even though numerous genomes exist for the family Pieridae, this is the first chromosomally complete genome sequence for the endemic species to Corsica and Sardinia, Euchloe insularis (Staudinger, 1861). Moreover, its taxonomic status was only very recently clarified (Wiemers et al. 2020). Vicariant endemic species for two major European islands ask for special attention from the communities of conservationists and taxonomists, so it is a valuable contribution to advance our scientific knowledge by knowing its complete genome. What I find very peculiar is that this species has 32 chromosomal pseudomolecules, while the majority of Pieridae species have n=31.  This is further proof of a separate taxonomic status of this species. We can only speculate at this stage about where this 32nd chromosome arrived. Is it a split of part of the original W chromosome and fusion with Z? As I see from Table 3, such a scenario is probable. Only studying males of *Euchloe insularis *and closely related species could reveal additional evidence on chromosomal rearrangements within this taxon. Since this species is not rare in the locality of Novella, Corsica, my suggestion is to also study the chromosomally complete genome of males and closely affiliated other Pieridae species. Using this opportunity, I would recommend that the authors indicate Corsica in the Sample acquisition subchapter, since it is not mainland France.

Are the datasets clearly presented in a usable and accessible format, and the assembly and annotation available in an appropriate subject-specific repository?

Yes

Are sufficient details of the sequencing and extraction, software used, and materials provided to allow replication by others?

Yes

Are the rationale for sequencing the genome and the species significance clearly described?

Yes

Are the protocols appropriate and is the work technically sound?

Yes

Reviewer Expertise:

Taxonomy, Lepidoptera, Karyology, Bioinformatics

I confirm that I have read this submission and believe that I have an appropriate level of expertise to confirm that it is of an acceptable scientific standard.

# Reviewer report: Reviewer response for version 1

**Published:** 2026-09-14

**Authors:** Automated Benchmarking

**Affiliations:** 1 Wellcome Sanger Institute, Hinxton, UK

As this article is a Genome Note, Automated Benchmarking has been used to determine whether the reported assembly passes three defined checks established by the Earth BioGenome Project: the raw and assembled data are publicly available, the Earth BioGenome Project three-part metric is met, and the BUSCO score passes the stated threshold. For Euchloe insularis (ilEucInsu1), all three checks passed. Data available: pass. ENA study PRJEB82391, raw-read accessions ERR13957082 and ERR13947556, and assembly accessions GCA_964605045.1 and GCA_964605135.1 are public. Earth BioGenome Project three-part metric: pass. Haplotype 1 is reported as 6.C.Q66, meeting the 6.C.Q40 reference standard. BUSCO: pass. Haplotype 1, which is proposed as the reference assembly for this species, has 99.0% complete BUSCOs (98.8% single-copy and 0.2% duplicated). BUSCO software version: 5.5.0; lineage dataset: lepidoptera_odb10. It meets the target of greater than 90% complete and single-copy BUSCOs. This decision uses values reported in the Genome Note and public accession records.

Reviewer Expertise:

NA

I confirm that I have read this submission and believe that I have an appropriate level of expertise to confirm that it is of an acceptable scientific standard.
